# Diagnosis of incidental gallbladder cancer after laparoscopic cholecystectomy: our experience

**DOI:** 10.1186/1471-2482-13-S2-S20

**Published:** 2013-10-08

**Authors:** Alessia G Ferrarese, Mario Solej, Stefano Enrico, Alessandro Falcone, Silvia Catalano, Giada Pozzi, Silvia Marola, Valter Martino

**Affiliations:** 1 University of Turin - Department of Oncology - School of Medicine -Teaching Hospital "San Luigi Gonzaga" - Section of General Surgery - Orbassano - Turin

## Abstract

**Background:**

Gallbladder carcinoma is a rare high malignancy neoplasm. The incidence of intra or post-operative incidental gallbladder carcinoma diagnosis is estimated between 0,2 and 2,8%. Primary aim of our study is to evaluate incidental gallbladder carcinoma's incidence in our experience.

**Methods:**

We retrospectively reviewed our Surgery Division's experience about the totality of laparoscopic cholecystectomies with post-operative histological evidence of incidental gallbladder cancer. We evaluated patients' characteristics, surgical related variables, histological response, surgivcal radicalization characteristics and surgical outcome.

**Results:**

In the considered sample we observed 7 accidental gallbladder adenocarcinomas in post-operative histological examination. Pathological results were:1 pT1b N0 (G1), 2 pT2 N0 (G2), 2 pT2 N1 (G3b), 2 pT3 N1 (G3b) (Table 1). In 5 cases we performed neoplasm radicalization surgery with standard procedure revision. Two patients died before radicalization. Median global survival was 34 months.

**Conclusion:**

With the increase of laparoscopic cholecystectomies both elective and urgent performed in our centre we observed also an increase of incidentally diagnosed gallbladder neoplasms. Early diagnosis, meticulous peri-operative study and accurate surgical strategy are essential factors to obtain good results in incidental gallbladder cancer.

## Background

Gallbladder carcinoma is a rare, high malignancy neoplasm, with an incidence rate of 0,3-1,5%[1-3]. The incidence of intra or post-operative incidental gallbladder carcinoma diagnosis is estimated between 0,2 and 2,8%; in this group, 15-30% of patients prove to be asymptomatic at the time presentation, without clinical evidences, intra or pre-operative, of neoplasm [1-3]. Primary aim of our study is to evaluate incidental gallbladder carcinoma's incidence in our experience, and to correlate it with age variable; second end-point of the study is to consider the possible relation between laparoscopic cholecystectomy indication increase in our centre and neoplasm observation incidence.

## Methods

Our work is a retrospective study conducted at University Section of General Surgery in "San Luigi Gonzaga" Hospital, Orbassano (Torino). We reviewed our Surgery Division's experience about the totality of laparoscopic cholecystectomies between November 2008 and November 2012. Afterwards we created a subgroup (N Group - Neoplasm Group), made of patients who received a post-operative histological diagnosis of gallbladder neoplasm. In this group we studied: demographic variables (age, gender, ethnic group), clinical variables (clinical presentation, symptoms), haematic values (WBC, CRP, bilirubin), pre-operative ecographic features (associated cholelythiasis, gallbladder adenomyomathosis, cholecystitis, evident hepatic lesions), intra-operative data (type of surgery, elective or urgent surgery, intra-operative complications), histology (stage, grade, resection margins), and surgical outcome (radicalization technique, result), adjuvant chemotherapy (Table 1) [3,4,10-14]. We excluded from the study all patients with pre-operative malignancy suspect.

**Table 1:** Data about demographic and operative characteristics

Pt.n°	Sex	Age (yrs)	Clinical presentation	Laboratory Data	US Data	1^st ^Surgical procedure	I.O. Gallbladder Perforation	2 ^nd ^Surgical procedure	Stage	Margin Resection	Adiuvant CT	Outcome
1	F	64	Normal	Normal	Cholelythiasis	OLC	No	-	pT3N1	-	-	Dead
2	F	64	Abdominal pain	WBC 12.8 PCR 15.43 Bil nn	Cholelythiasis	ELC	No	Radicalization	pT2N0	R0	Yes	
3	M	63	Normal	Normal	Adenomiomatosis	OLC	No	Radicalization	pT1bN0	R0	No	
4	M	70	Abdominal pain	WBC 12.7 PCR 29.72 Bil nn	Adenomiomatosis	ELC	No	Radicalization	pT2N1	R1	Yes	Dead
5	F	75	Abdominal pain	WBC 9.67 PCR 3.41 Bil nn	Cholecystitis	ELC	No	Radicalization	pT2N0	R0	Yes	
6	M	74	Normal	Normal	Cholelythiasis	OLC	No	Radicalization	pT2N1	R1	Yes	Dead
7	F	65	Normal	Normal	Cholelythiasis	OLC	Yes	-	pT3N1	-	-	Dead

For stadiation we used the Sixth Edition of AJCC TNM Manual. All laparoscopic cholecystectomies were executed with standard 4 trocars thecnique, and Endo-Bag protected gallbladder extraction.

In the matter of surgical radicalization, we executed 4b-5 segmentectomies (we did not perform right hepatectomies) [6,7]. We always performed hepatic peduncle lymphadenectomy: if peduncle's nodes resulted positive, we executed N1 lymphadenectomy with hepatic porta and over-duodenal nodes removal. If second station nodes were suspected, we executed additional lymphadenectomy.

Resection completeness was classified in: R0 without residuals on hepatic margins, R1 microscopically positive margin, R2 macroscopic residuals on hepatic margins.

## Results

We analyzed 508 consecutive laparoscopic cholecystectomies: 457 executed for cholelytiasis (150 urgencies), 51 patients for gallbladder adenomyomathosis (all elective).

We observed 7 accidental gallbladder adenocarcinomas in post-operative histological examination (N Group - Neoplasm).

4 patients out of 7 were females (Table 1), and the mean age of Group N was 67,8 years old (range 64-75). The totality of the sample resulted in Caucasic ethnic group.

Three cholecystectomies were performed in emergency (2 for acute lythiasic cholecystitis with US evidence, and 1 for alythiasic cholecistitis in adenomyomathosis) and the other 4 in elective regime (1 for adenomyomathosis, 3 for cholelytiasis) (Table 1). Patients who underwent urgent surgery presented acute inflammatory haematic setting (Table 1). Hepato-biliary settings at admittance were normal in all patients. Intra-operative gallbladder wall morphology appeared normal in all cases. Cholecistectomies were all carried out by laparoscopy without laparotomic conversions. Gallbladder was always extracted with protections, and we didn't observe cutaneous trocar-site metastases.

Pathological results were:1 pT1b N0 (G1), 2 pT2 N0 (G2), 2 pT2 N1 (G3b), 2 pT3 N1 (G3b) (Table 1). In 5 cases we performed neoplasm radicalization surgery with standard procedure revision: hepatic 4b-5 segmentectomy, hepatic peduncle lymphadenectomy and trocar-site excisions. We didn't perform radicalization right hepatectomies. We executed a N1 lymphadenectomy, and the second station resulted intra-operatively negative. Two patients died before radicalization (both patients with pT3N1 histological finding). We didn't observe any intra-operative biliary ducts invasion, and we didn't perform any biliary resection.

Pathological results on hepatic margin were: 3 R0 and 2 R1 (these two both on pT2N1 histological result and both patients died in 1 month). 4 patients underwent adjuvant chemotherapy (Table 1).

Median global survival was 34 months; 3 patients are still alive and, at now, disease free.

## Discussion

In literature gallbladder cancer incidence is 0,3-1,5%; it also seems to be an increase over last years. In our study incidental gallbladder adenocarcinoma's rate resulted 1,38%. Some authors affirm that major risk factors for the disease are: female gender, obesity, age over 60 years old, and cholelythisasis [3,4,10-14]. According to literature, incidence resulted higher in female gender (4/7 patients), in patients with a long story of cholelythiasis (5/7), and the whole sample resulted aged over 60 (7/7). Symptoms are aspecific and the most important prognostic factor is pathological stage; also our patients resulted asymptomatic for the questioned disease [3,4,10-14].

Literature reported median survival rate varies between 8,1 and 68 months (range 3-100 months); in our study median survival was 34 months (range 2-63 months).

Surgical resection with curative intent in post laparoscopic cholecystectomy gallbladder carcinoma is: for stage T1a surgery is not proved to be necessary but watchful follow-up only seems to be required, for stage T1b the correct approach is still debated and some authors define as sufficient hepatic gallbladder bed resection with hepatic peduncle lymphadenectomy, for stage T2-3-4 surgical approach with hepatic S4b-5 radicalization (or more extended, "a la dèmande") is recommended, with hepatic peduncle lymphadenectomy associated to trocar-site excisions [4,5,8,11,12].

Some authors from Sloan-Kattering Institute, basing on their experience and cases, drew up a flow-chart for a correct re-evaluation method in incidentally diagnosed gallbladder cancer, suggesting always to perform an explorative laparoscopy at the beginning of radicalization surgery (that must be carried out by open technique), to exclude peritoneal carcinosis; in this way there is the possibility to better identify patients eligible for laparotomic radicalization [8].

## Conclusion

Incidental gallbladder cancer incidence in literature is reported with the higher value of 2,85% (biblio); in our experience this rate resulted to be 1,38%. Despite retrospective study limits and the small N Group sample, with the increase of both elective and urgent laparoscopic cholecystectomies perfored in our section we observed also an increase of incidentally diagnosed gallbladder neoplasms (Table 2) [15,16]. Surgery is more difficult in elderly patinents [17] but we consider laparoscopy as feasible and secure technique also in the elderly [18]. According to literature, also in our experience incidental gallbladder cancer resulted more frequent in over 60 years old patients and in female subjects. We consider being fundamental the extemporaneous histological test during cholecystectomy in case of intra-operative disease nature doubt. Gallbladder cancer mortality remains high for its biological aggressiveness [9-11]; surgical treatment is still the only radical treatment possibility. Also in our experience early diagnosis, meticulous peri-operative study and accurate surgical strategy are essential factors to obtain good results in incidental gallbladder cancer.

**Table 2:** Data about years stratification.

Years	TOT VLC	GBc
2008	81	0
2009	97	0
2010	107	3
2011	111	3
2012	112	3

TOT - VLC: Total videolaparocholecystectomy performed, GBc: Incidental Gallbladder Carcinoma

## Competing Interests Statement

The authors declare that they have no competing interests.

## Authors' contributions

AGF: conception and design, interpretration of data, given final approval of the version to be published.

SE: conception and design, interpretration of data, given final approval of the version to be published.

MS: acquisition of data, drafting the manuscript, given final approval of the version to be published.

AF: acquisition of data, drafting the manuscript, given final approval of the version to be published.

SC: acquisition of data, drafting the manuscript, given the final approval of the version to be published.

GP: acquisition of data, drafting the manuscript, given the final approval of the version to be published.

SM: critical revision, interpretation of data, given final approval of the version to be published

VM: critical revision, interpretation of data, given final approval of the version to be published

## Declarations

Funding of this supplement has come from personal funds.

This article has been published as part of *BMC Surgery *Volume 13 Supplement 2, 2013: Proceedings from the 26th National Congress of the Italian Society of Geriatric Surgery. The full contents of the supplement are available online at http://www.biomedcentral.com/bmcsurg/supplements/13/S2
